# *Have my back as I get back to work*—Experiences of stakeholder support in returning to work after sick leave due to chronic pain: A qualitative interview study

**DOI:** 10.1371/journal.pone.0312478

**Published:** 2024-10-23

**Authors:** Åse Lundin, Inger Ekman, Paulin Andréll, Mari Lundberg, Sara Wallström

**Affiliations:** 1 Institute of Health and Care Sciences, Sahlgrenska Academy, University of Gothenburg, Gothenburg, Sweden; 2 University of Gothenburg Centre for Person-Centred Care (GPCC), Sahlgrenska Academy, University of Gothenburg, Gothenburg, Sweden; 3 Department of Medicine, Geriatrics and Emergency Medicine, Sahlgrenska University Hospital/Östra, Gothenburg, Region Västra Götaland, Sweden; 4 Department of Anaesthesiology and Intensive Care Medicine/Pain Centre, Region Västra Götaland, Sahlgrenska University Hospital, Gothenburg, Sweden; 5 Department of Anaesthesiology and Intensive Care Medicine, Institute of Clinical Sciences, Sahlgrenska Academy, University of Gothenburg, Gothenburg, Sweden; 6 Department of Health Promoting Science, Sophiahemmet University, Stockholm, Sweden; 7 Department of Forensic Psychiatry, Region Västra Götaland, Sahlgrenska University Hospital, Gothenburg, Sweden; 8 Centre for Ethics, Law and Mental Health (CELAM), University of Gothenburg, Gothenburg, Sweden; University of Pretoria, SOUTH AFRICA

## Abstract

**Background:**

Chronic pain (pain > 3 months) is a disabling condition affecting around one fifth of the population. Chronic pain significantly affects a person’s psychological and physical health and often interferes with the ability to work. It is one of the most common reasons for extended sick leave and persons with chronic pain often have difficulties returning to work. Interpreting the experiences of currently available is necessary in order to facilitate a return to working life. Therefore, this study aimed to describe and interpret the meaning of support during the return-to-work process for persons on sick leave due to chronic pain.

**Method:**

A qualitative interview study was conducted with 14 participants (12 women and 2 men) who experienced sick leave due to chronic pain. The participants were recruited through patient organizations focusing on pain or pain-related conditions. Collected data was analyzed using a phenomenological hermeneutical approach.

**Results:**

*Have my back as I get back to work* was the theme of the analysis, along with six subthemes. Being able to work was important for the participants. However, they often experienced returning to work was a battle for support, dealing with fragmentized backing from the involved stakeholders. Participants with access to collaborative support involving competent care, recognition and the possibility to influence their work felt valuable and capable as persons and workers. Thus, they were provided conditions allowing a successful re-entry into the workplace.

**Conclusions:**

Our findings contribute to an enhanced understanding of the importance of stakeholder support in persons with chronic pain re-entering the workplace after an extended break due to sick leave. Through an inclusive, collaborative and flexible approach involving all stakeholders working towards the same goal, a person with chronic pain can feel supported in developing and cultivating the capabilities necessary to manage life and work.

## Introduction

Chronic pain affects about a fifth of the population [1,2] and is a leading cause of years lived with disability worldwide [3]. It is defined as pain that persists or recurs for over 3 months [4] and it significantly affects a person’s physical and psychological health and well-being [5]. Chronic pain is associated with mental health issues such as depression [6], increased risk for substance abuse [7], elevated risk of suicidal ideation and suicide attempts [8] and reduced life expectancy [9]. Affected family life, social relationships, and financial hardships are common consequences of chronic pain [10]. Chronic pain often interferes with the ability to work. In Sweden, it is the second most common reason for being on long-term sick leave [11]. According to the European Working Conditions Survey, persons with chronic pain miss three times as many days of work per year as those without chronic pain [12]. The societal costs of chronic pain are extensive, with the main component relating to absence from work [13–15].

Interventions and strategies to facilitate return to work (e.g., part-time sick leave or graded sick leave certificates, early ergonomic interventions and disability evaluation followed by information and advice) have shown positive outcomes for sick leave and return to work in persons with chronic pain [16]. Multimodal rehabilitation programs, comprising physical and psychological components and work-targeted approaches, can increase the likelihood of persons with chronic pain continuing in the workplace [17]. Vocational rehabilitation, which aims to optimize work participation, is also common among persons with health-related impairments, limitations or restrictions in work functioning [18]. However, evidence for tertiary interventions on returning-to-work with chronic pain remains inconclusive [19] and it is still widespread for persons with chronic pain to have difficulties returning to work despite interdisciplinary interventions [20].

Previous qualitative research has reported what persons on sick leave due to chronic pain consider important aspects in the return-to-work process. Workplace modifications, adaptions to individual needs [21–23], having a strong collaboration among different stakeholders throughout the rehabilitation [21,24] and good communication with employers and colleagues [25] have been identified as critical supportive experiences in returning to work. Studies have also described several barriers to persons re-entering the workforce, including employers’ limited understanding and awareness of chronic pain [26], the unpredictability of pain and inflexible work environments [27], the negative impact of organizational systems [28] and barriers to receiving health care and the inefficiency and ineptitude of the healthcare system [26,29].

While qualitative approaches are accelerating within chronic pain research, most studies incline towards descriptive analysis, thus missing out on a more profound engagement with participant experiences [30]. There is considerable explanation, however limited research on interpretation of the essential meaning of support for persons with chronic pain when going back to work. Diving deeper, beyond description and towards the essential meaning of lived experience, is of crucial importance in demanding caring situations [31]. To be able to understand and improve our practice, we have to take part and be moved by narration to attain understanding [31]. An increased understanding of the experiences of stakeholder support is fundamental to appreciate these persons’ needs and facilitating a return to working life. Therefore, this study aimed to describe and interpret the meaning of support during the return-to-work process for persons on sick leave due to chronic pain.

## Methods

### Design

A qualitative research design was chosen to explore participants’ experiences of support upon returning to work after sick leave. We conducted semi-structured interviews using open-ended questions. The interviews were analyzed using a phenomenological hermeneutical method developed by Lindseth and Norberg to investigate lived experiences [31]. The technique is inspired by Ricoeur’s theory of interpretation [32] and seeks to understand the meaning of participants’ lived experiences by interpreting their narrations.

Standards for Reporting Qualitative Research (SRQR) guidelines have been followed to ensure trustworthiness in reporting the findings [33].

### Participants and setting

Participants were recruited from all over Sweden, as the possibility to participate through digital channels did not require the participants to live close to the University of Gothenburg premises. A purposeful sampling strategy was used with the aim to reach a diverse setting of participants with variation in age, gender, educational background and pain location, in order to acquire a wide array of experiences of sick leave due to chronic pain. Information on the study was sent to patient organizations and associations focusing on pain or pain-related conditions who distributed a request to their members through e-mail, newsletters or Facebook pages. The inclusion criteria were 1) all participants must be ≥18 years of age, 2) have previous or current experience of being on part-time or full-time sick leave from work due to chronic pain (pain duration >3 months), 3) speak Swedish and 4) willingness and ability to participate in an interview on one occasion.

### Data collection

The first author (ÅL) conducted semi-structured in-depth interviews between January and August 2021. The interviews lasted 41–90 minutes and were audio recorded on dictaphone and subsequently transcribed verbatim. Participants chose the location for the interview. Four participants were met in person: two on the university´s premises and two in the participants’ homes. The remaining 10 interviews were conducted through digital channels (e.g., Zoom), and only audio was recorded, that was later transcribed verbatim as well. The transcribed audio material amounted to just above 230 pages of text, this text forming the basis for the material used in the analysis.

At the start of the interviews, participants were informed of the study’s aim and were encouraged to raise questions or concerns. An interview guide was used (S1 File). Participants were asked guiding questions, such as ‘*In what way would you say that your chronic pain has affected your work situation*?*’*, ‘*What support have you needed to deal with your situation*?*’*, *‘Do you feel that you have received such support or adjustments at your workplace*? Probing follow-up questions were asked to encourage further narration: *‘What has that meant to you*?*’* and ‘*How did that make you feel*?’.

### Analysis and interpretation of data

The analysis in phenomenological hermeneutics involves a dialectical movement between three stages: *naïve reading*, *structural analysis* and *comprehensive understanding*. The analysis started once all the interviews were completed. In the *naïve reading* stage the text was read several times to get a first impression and grasp its meaning as a whole. From this analysis, a naïve understanding was formulated. In the *structural analyses* the text related to the study’s aim was divided into meaning units. A unit could consist of a few words, a sentence or even a paragraph with one essential meaning. The meaning units were read through, reflected upon and sorted for similarities and differences, then grouped into subthemes and a theme. The structural analysis is methodological and often lies close to the text; therefore the themes are not formulated as abstract concepts, but instead as condensed descriptions that discloses meaning [31]. During the structural analysis, all texts were considered, but the parts not related to the aim were not included in the formulation of themes. Software for qualitative data analysis (QSR NVivo R1) was used as a sorting tool in the structural analysis.

Lastly, a *comprehensive understanding* was formulated. It contains a dialectical movement between all stages of analysis, between the parts and the text as a whole, as we try to arrive at a deeper understanding and disclose new possibilities for being in the world [31]. The entire text was reread with an open mind. The naïve understanding and the validated subthemes and theme were discussed and reflected on in relation to the research question, the authors’ preunderstandings and the study context. Relevant literature was used to illuminate the text and widen the horizon of interpretation [31]. ÅL, IE, SW, ML and PA met regularly throughout all stages of the analysis to discuss the naïve understanding, emergence of the subthemes and theme and comprehensive whole. This approach provided different perspectives and ensured a congruent understanding and interpretation of the text.

### Ethical considerations

The study was approved by the Swedish Ethical Review Authority (Ref. 2020–02491). All participants received oral and written information about the study, and verbal and written informed consent was obtained from each participant before study participation. To safeguard participants’ identity all collected data—text and audio—were made confidential through numerical coding and stored safely using a multi-factor authentication method. The study was conducted in accordance with the principles of the Declaration of Helsinki [34].

### Research characteristics and reflexivity

ÅL is a physiotherapist with several years of clinical experience in primary health care and thus regularly encounters patients experiencing chronic pain. ÅL has experience in qualitative pain and mental health research. Of the four co-authors, three have extensive experience working with qualitative analyses (SW, IE, ML), and two have vast experience in rehabilitation and research on chronic pain (ML, PA). None of the participants were known to the researchers before the start of the study. During the study’s initial phase, the first author, in writing and discussion with the co-authors, reflected upon the pre-understandings (set of prior assumptions, beliefs and attitudes) in the field of chronic pain, the research questions and methodological approaches and how these pre-understandings may influence the research.

## Results

Eighteen persons responded to the participation request. Of those 18, three decided not to go ahead with an interview and one did not attend the booked interview session. Thus, the final sample comprised 14 participants. The majority of the participants were women, had attended university or other upper secondary education, and had lived with chronic pain for more than 10 years.

Participant characteristics are summarized in Table 1.

**Table 1:** Participant characteristics.

Men/Women	2/12
Mean age, years (range)	49
	(23–80)
*Highest completed education*	
Primary school (9 years)	2
High School	3
Other upper secondary education	4
University degree	5
*Pain condition*	
Fibromyalgia	5
Ehlers-Danlos syndrome	4
Spinal injury	2
Neck/shoulder pain	1
Rheumatic disease	2
*Duration of pain*	
1–3 years	2
4–9 years	1
>10 years	11
*Current work status*	
Full-time sick leave	4
Part-time sick leave	5
Returned to work	4
Retired	1

### Naïve reading

The naïve reading of the text indicated that going back to work while living with chronic pain was a double-edged sword. Participants strongly identified with working and longed to return to a meaningful work experience in which they feel empowered to contribute significantly to their families and society. However, because participants experienced a lack of stakeholder support, they were concerned about whether returning to work was a realistic goal. Lacking support early on in the process often left participants feeling alone on the battlefield of pain and anxiety. They felt a need for an increased understanding of the difficulties of chronic pain and support from the stakeholders (e.g., employers and colleagues, the social insurance system and the healthcare system) involved in the return-to-work process. Without acknowledgment and support from others, participants did not know whether work and pain were compatible, creating additional stress and worry for the future.

### Structural analysis

**Fig 1:**
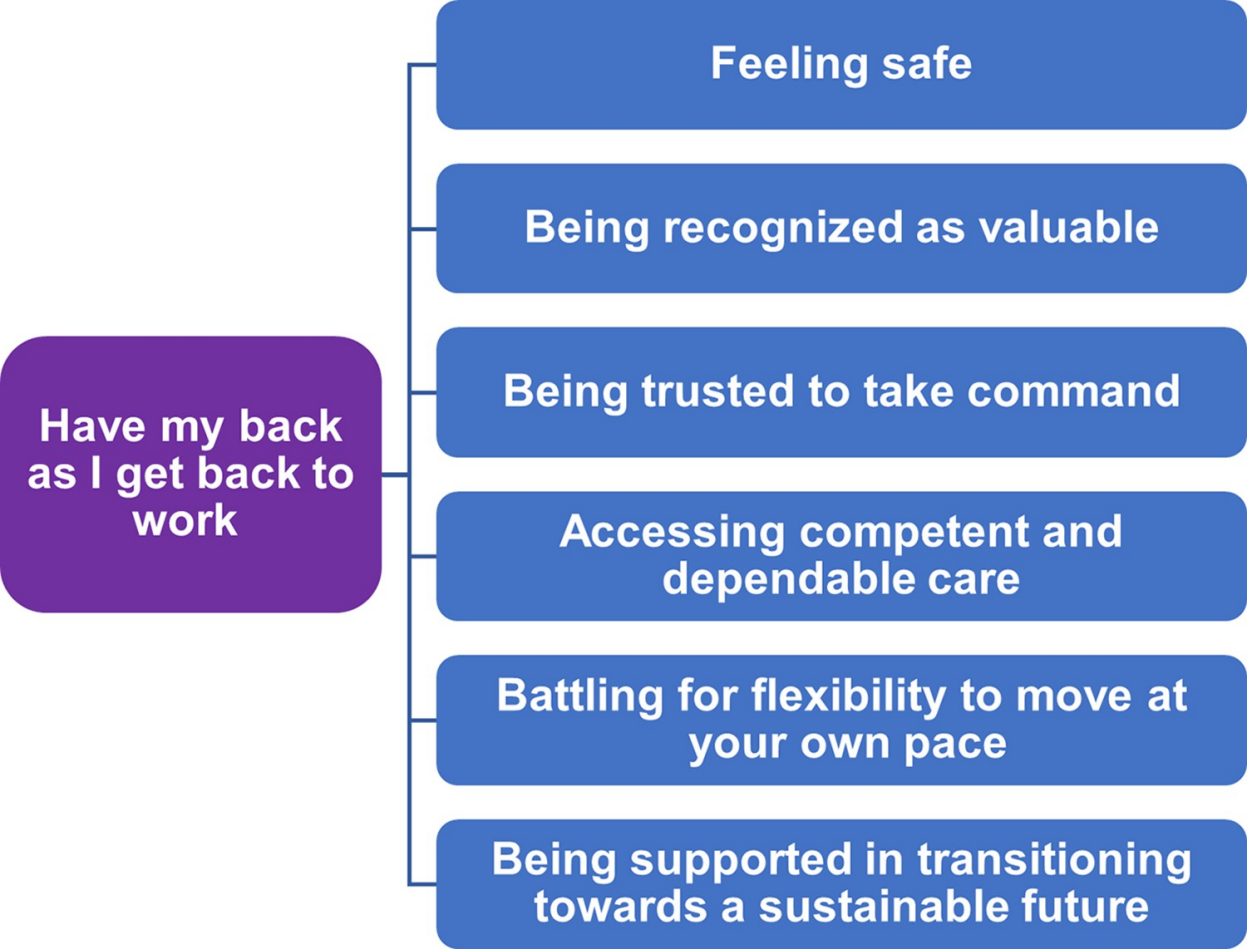
Overview of the theme and six subthemes.

The structural analysis resulted in one theme and six subthemes (Fig 1) and described in-depth below.

#### Theme: Have my back as I get back to work

Participants faced many obstacles returning to working life while coping with the consequences of chronic pain. It was often a lonely process plagued by feelings of vulnerability, incapability and worthlessness. In addition, participants were worried and stressed about the future and its uncertainties. To overcome these obstacles participants expressed the need for dependable and cooperative support from stakeholders during their return to work. It was important for participants to receive support from their employer, healthcare system, social insurance and employment services. Having someone’s back means watching out for someone´s well-being. It represents a trustful relationship built on collaboration and collective responsibility where the stakeholders strive to support participants in re-engaging with work. Participants still had to work hard and be the driving force in their rehabilitation and re-entrance into the workforce. As in any battle, participants knowing there were reliable allies was crucial to facilitating the conditions necessary to successfully re-enter the workplace.

#### Subtheme 1: Feeling safe

Participants struggled to be open about their pain at their workplace. Disclosing their health issues meant being laid bare, and also exposed to the risk of distrust or delegitimization. There was an anticipation or fear of stigma and that revealing their illness could have adverse consequences on relationships at the workplace. Feelings of vulnerability and the fear of consequences made them hesitant to reveal their condition and thus be perceived by their employer as cumbersome or demanding. In contrast, being met with understanding, empathy and responsiveness from managers and colleagues provided a safe psychosocial environment where participants felt acknowledged and secure to be open about their illness.

*But now I also have a manager who I have been able to confide in and share my situation, and it’s damn tough, damn tough. I have a great manager right now, and I’m grateful for that; someone who thinks about these things, is aware of the situation, responsive and listens. So that feels good! But I think it’s not easy to confide in someone…you expose yourself and don’t do that to just anyone. #6*

#### Subtheme 2: Being recognized as valuable

Participants often pushed themselves hard to return to work because it was seen as a source of joy and well-being. They saw themselves as dutiful workers and wanted to be appreciated for their work ethic. Therefore, it was important to feel supported through recognition as a valuable and attractive asset instead of a burden or liability. Because participants were deeply invested and committed to their job, they wanted their employers to consider them worthwhile. Participants acknowledged that they differed from before they became ill and might not perform at the same level, due to consequences of the pain, such as fatigue. However, there was still a wish to be viewed as a capable person and a valuable contributor at their workplace as well as be recognized for former contributions. One participant described the disappointment and feeling of injustice after working for many years for the same employer when, after being on sick leave for a year, the employer saw no future value in having her employed and pressured her to resign.

*Nobody bets on a horse that doesn’t win, but I have won so many times in the past…//…as much effort that I’ve put in and as much I’ve helped others and supported and been a good colleague…#3*

#### Subtheme 3: Being trusted to take command

Participants who could influence their work environment saw this as a significant contributing factor to a successful return to work. Workplace adjustments regarding tasks, workload, physical aids and work environment gave participants a strong sense of value as a person beyond being a mere employee. Finding tailored solutions in the workplace required a joint effort and a dialogue with the employer, and participants put enormous value in being allowed to assume control over their work. Creating their work schedule according to pain fluctuations gave participants a sense of acknowledgment and a feeling that the employer has confidence in their ability to perform well.

*I have a job that I can organize depending on how I feel. So, if I have a terrible morning, I can catch up on that work in the afternoon and evening, and in general, I have noticed over the years that the best way for me is to work two full days and a half day so that I get four days of recovery. And my employer allows me to do that…It has meant a lot because it shows they have confidence in me. That I do the hours that I’m supposed to do. #12*

#### Subtheme 4: Accessing competent and dependable care

The actions of the healthcare sector were vital to participants’ well-being and whether they succeeded in returning to work. Having dependable and competent support from health care professionals and access to treatment such as pain rehabilitation helped participants develop strategies to balance going back to work, everyday life and the obstacles caused by pain. However, participants often experienced a lack of resources and organizational barriers within the healthcare system. Experiencing a lack of competence on chronic pain and stigmatic attitudes when meeting healthcare personnel was also common. Not being met with attentiveness and understanding further decreased participants’ well-being and made them hesitant to seek further care. There was also a desire for increased collaboration between the healthcare sector and the workplace as regards their rehabilitation.

*Oh yes! If I hadn’t had this doctor…it probably wouldn’t have been that good. It has also been security that I know I have a good doctor to always turn to, who listens. After all, she has also followed me for all these years, has been with me on my journey, and still is. So, I think the safety of having your doctor is important. #6*

#### Subtheme 5: Battling for flexibility to move at your own pace

Several participants felt stressed about returning to work because they were unprepared or not in control of their pain. They often experienced that the Social Insurance Agency or the employment services forced them back to work before they were ready. Feeling pressured to return to work could be linked to the rigid and inflexible policies and regulations on approving sick leave. The absence of flexibility was perceived by the participants as a battle to be allowed to be absent from work and rehabilitate while ill. This conflict often caused additional mental wellness issues, such as anxiety and stress. Sometimes participants did not have the strength nor the energy to stand up for themselves, losing dignity and feeling defeated. In contrast, participants that were met with an understanding of the complexity of their condition and flexibility in the system felt acknowledged and allowed to keep a more manageable pace, yielding increased well-being in their return process.

*Of course, living with something like this is stressful when you realize I’m not going to, or I can’t work and need time. I want to go back, but I need time. But they won’t listen to that. And, of course, that becomes an additional stress that affects me negatively. #7*

#### Subtheme 6: Being supported in transitioning towards a sustainable future

Participants wanted to continue working, but there were concerns about working in the future under conditions of chronic pain. Several participants looked at other jobs or re-education to find a sustainable work environment. Being burdened by chronic pain and, at the same time, having to make decisions about their future work and career was a lonely and daunting process. Because of feelings of exhaustion and being worn out from their pain, participants had limited ability to lead this transition independently. There was a need for extensive guidance and support to find suitable work options.

*Because it is not easy being in a completely new situation, sitting in a wheelchair, going out onto the job market and trying to look for a job. And I used to drive a truck, which doesn’t work anymore! Should you then start re-training and things like that… it’s not easy! #1*

*I do have a chance to change my life*, *career-wise*. *But I don’t have the energy for anything at all*…*//*…*And*, *of course*, *it would feel easier to change jobs*, *but it’s not just to change your career*! *I can’t do anything and am not receptive to any information*. *I’m so exhausted*!…*//…I don’t have the energy*. *I…I’m going to have to start an education or*…*also*, *I don’t have a driver’s license*, so that means the job opportunities are cut in half *sighs* #3

### Comprehensive understanding

Through dialectical movement, the naïve reading, structural analysis and our pre-understanding were considered, as well as the use of associated literature to illuminate our interpretation further and gain a comprehensive understanding of the text. The authors accessed literature on the *capability approach*, a framework based on functioning and capability pioneered by the economist and philosopher Amartya Sen [35] and developed by Martha Nussbaum, Vikki Entwistle and Ian Watt [36,37] among others. Nussbaum’s text *“Creating Capabilities*” [37] focuses on a dignified human life in which persons have the capability to achieve lives they value. Capabilities are not solely internal or due to personal characteristics but are possibilities created by personal characteristics combined with social, political and economic circumstances [37]. Entwistle and Watt developed the importance of relational factors and recognized that capabilities are dynamically shaped by interactions between individuals and their environment, including social relationships [36].

Building upon those recognitions, the authors believe that for participants to return to work, they need to feel others support their capabilities. Such support requires a joint effort involving all stakeholders and the participants’ environment. The capability approach is closely related to ideas of health, human rights and social justice [38] and the theme and subthemes were looked upon in relation to Nussbaum’s central capabilities [37]. Justice was related to the participants’ need for access to competent care and having the right to fair, respectful and non-stigmatic encounters with the healthcare system, the social insurance services and the workplace. It is also a question of justice and health in having support to find work. In the workplace persons can form meaningful and affirmative relationships based on dignity and mutuality. Participants battled for a safe workplace environment, both psychologically and physically, and regarding adjustments and being allowed to influence their work situation. This environment depended greatly on the efforts, understanding and responsiveness of their managers and colleagues.

Inspired by the capability approach, the authors expanded the interpretation that the meaning of support when returning to work entails support from others in developing and cultivating the capabilities of those with chronic pain. We refer to this as *Have my back as I get back to work*, which can be regarded as the main theme. To have someone’s back represents a trustful relationship built on collaboration and collective efforts, where stakeholders work together while letting those returning to work take responsibility for their rehabilitation. Being able to work was necessary for participants to achieve lives they value, but they often had to struggle to attain this goal. What participants wanted was for stakeholders to provide trustful support and encouragement during challenging times, e.g., to have their back in this battle.

## Discussion

The results of this study contribute to an enhanced understanding of the meaning and importance of having supportive and enabling relationships and environments for persons with chronic pain returning to work. Cultivating and strengthening a person’s capability is essential to reintegrating persons into the workplace. However, it requires acknowledgment of their value and stakeholder collaboration. Being backed by stakeholders (mainly the employers, the healthcare system and the social insurance authorities) is experienced as a decisive factor in achieving capability and feeling acknowledged and safe.

Many of our participants experienced a lack of collaborative support. The supportive actions that stakeholders provided were fragmentized, obstructing the rehabilitation effort. Previous research describes similar difficulties in managing all the different contacts with stakeholders throughout the return-to-work process [21]. Moreover, a lack of collaboration or clear links between stakeholders can lead to a feeling of being passed around or falling between the cracks [21]. Our results show a need to work together to find common goals and facilitate the means to reach them. Reconciliation meetings, in which the different perspectives of the employee, the healthcare system, the employer and the policy representatives are all considered and intertwined [24], is one way to ensure that stakeholders share goals and expectations [21]. Previous qualitative research supports reconciliation meetings and stakeholders’ involvement and collaboration in facilitating a return to work after an extensive leave [28]. Entwistle and Watt state that person-centered care is an approach that recognizes and cultivates capabilities, thereby acknowledging that a person’s capability is relational rather than individualistic and that capabilities are exercised and developed in relationships with others [36]. There is no universal set of rules for persons returning to work with chronic pain. Building and working in a partnership includes sharing information, deliberation and decision making, and are all key components of person-centred care. A viable partnership must also consider the person’s lifestyle, preferences, beliefs, values and health issues [39]. The capability approach and person-centered care are rooted in an ethic concerning what living a good life entails [36].

Our participants viewed the managers as a key for them to successfully return to work as they had the opportunity (and responsibility) to make workplace adjustments. Additionally, managers and colleagues created an environment where participants could feel safe, affirmed and justly treated, all closely related to the central capability concept described by Nussbaum [37]. It also aligns with research showing that the employer is often the determining factor in whether a person succeeds in returning to work [40]. A lack of employer understanding of chronic pain could negatively impact the ability to return to work [27]. Therefore, it might be important to direct more expertise towards the workplace during rehabilitation and further extend the employers’ involvement in that they have a general view and competence about the work situation [25].

Stigmatic attitudes from their employers and disappointing or hostile encounters with the healthcare system were identified as barriers to feeling acknowledged and safe. Previous research shows that chronic pain is often associated with stigmatizing attitudes and reactions from the public, health care providers and family or friends [41,42]. Chronic pain-related stigma is also often experienced in the workplace, resulting in unsympathetic and sometimes belligerent encounters with employers or colleagues [43] or feeling ostracized [12]. The invisibility of pain and the lack of understanding of chronic pain contribute to the feeling of being met with distrust and suspicion, leading to a constant struggle for recognition and equitable encounters for a worker on sick leave [44]. Stigma can interfere with rehabilitation and hinder workplace reintegration [45]. Reducing stigma and creating safe, inclusive and cultivating environments is crucial in promoting the capability to return to work when suffering from chronic pain.

In contrast, when meeting health services, demeaning or undermining behaviors can destroy rather than cultivate a person’s capabilities [36]. This view is consistent with our results, as participants sometimes avoided seeking further health care or employer support because of the health care professional’s or employer’s cynical attitudes. Suggestions of interventions and strategies to reduce chronic pain-related stigma include education towards the public and health professionals [42,45], interventions on intrapersonal levels (e.g., enhancing resilience and coping among those targeted by stigma) and interventions on structural levels regarding laws, policies and institutions [46]. Some changes are underway. For instance, the International Classification of Diseases 11^th^ revision (ICD-11) proposes a coding system for chronic primary pain [47]. Acknowledging chronic pain as a legitimate disease and recognizing it in a systematic classification system is an opportunity to improve our understanding and management of chronic pain [48]. Increased legitimacy and reduced stigma faced in the encounters with stakeholders could offer a safer environment for participants and provide conditions for cultivating instead of hampering their capabilities.

Similarly, constantly battling for support when encountering impersonal and rigid social policies was identified as obstructing participants’ capabilities as it defeats one of the central capabilities; being in control over one’s environment [37]. In previous research this experience of losing influence while being governed by rules and regulations has been compared to a ball in a pinball machine, perpetually being exposed to unexpected decisions and changes without the possibility to control the situation [49]. Supporting capabilities is something that all democracies should aspire to [37]. Thus, the authors argue that regulations and policies in work and disability should be grounded in a capability perspective and person-centered approach. Being supported through sick leave and going back to work while having chronic pain can but does not have to be a lonesome endeavor. Instead, it can be a joint effort and a collaborative process based on teamwork and partnership where the person returning to work is acknowledged and affirmed.

A capability approach can aid in understanding and defining disability and formulating disability policies [50]. Looking at disability from a capability approach, disability can be understood as a deprivation of capabilities, which can result from impairment but also arise from barriers in resource availability or the social, economic, political and cultural environment [50]. Assessment and evaluation of capability within long-term health conditions are critical, as the emphasis is often to aid these persons to live a full life despite their chronic symptoms [51]. Employment is essential for persons with and without impairments and is a source of social participation and income [50]. Work is also an important domain of life in which values, goals and ambitions can be realized and contribute to health [52].

Contrary to studies reporting that some employers thought persons with chronic pain were “lazy” [27] or “work-shy” [45], the majority of participants in this study expressed a strong desire to return to work. However, they said they were hampered by the lack of supportive and collaborative actions to guide their return to work. In contrast, participants with access to support such as competent care, recognition and the possibility to influence the workplace felt valuable and capable as persons and workers. Most importantly, they were provided conditions allowing a successful re-entry into the workplace.

The capability approach emphasizes values, such as recognition and meaning, constituting an important aspect of work. How these values can be met are important considerations in reaching sustainability, employability and thus also health [52]. No man is an island. Only through being supported by enabling and facilitating environments, understanding and collaborative relationships with others can persons suffering from chronic pain develop their full capabilities.

### Methodological considerations and limitations

There are no strict guidelines for sample size and when to stop recruitment in qualitative research. However, sample sizes of at least 6 or between 5 and 25 persons are commonly suggested in phenomenological research [53,54]. Fourteen participants were included in this study. Obtaining deep and varied data is often considered more important than the sheer number of participants. An adequate sample size results in a new and richly textured understanding of an experience [55]. The authors regularly met during the collection and analysis of the data to assess their quality, richness and variation and to decide when sufficient narratives were reached to answer the research question. ÅL, IE, SW and PA were all involved in the analysis process to guarantee a congruent understanding of the data and increase trustworthiness.

One limitation of this study is that most participants were women. The most common cause of chronic pain in our study was fibromyalgia, a diagnosis more prevalent in women [56]. The sample characteristics might also be attributed to the recruitment method and a possibly higher activity or representation of women in the chosen patient organizations. Another limitation is that participants were required to speak Swedish to participate in the interviews, which excluded those not fluent in the language. This limitation should be considered when interpreting the results and during planning of future interview studies on the subject. The education level of participants was generally high, with most having upper secondary or university education. This higher education is reflected in participants’ line of work and job positions and thus also their opportunities of rehabilitation towards the labor market.

In designing the study the inclusion criteria did not contain an upper age limit to participate, which can be considered a limitation. The mean age of the participants was 49 years old, however one participant was 80 years old and had retired from the workplace. As both current and previous experiences of sick leave and returning-to-work was accepted the participant was deemed eligible to take part in the study. In hindsight we acknowledge that this participant provided valuable insights on lived experiences of the return-to-work process that would otherwise have been missed out upon. Data on the participants duration of sick leave was not collected, which is another limitation to this study. However the participants described various and different experiences including either long-term or shorter periods of sick leave, being on sick leave part-time or in full, as well as combination of these. We believe this brought a varied and rich perspective of the experiences of the studied phenomenon, hence can also be viewed as an advantage. Lastly, another consideration is that the focus of the interviews often tended to regard support from organizational levels, the healthcare system and the workplace. Future research should explore the meaning of support from other areas, including family, friends or peers. Studies should also investigate the effect of person-centered support when persons with chronic pain return to work.

## Conclusion

Returning to work after being on sick leave due to chronic pain was experienced as an unrelenting battle for collaborative stakeholder support. Interpreting the participants’ experiences enhanced our understanding of the will to–but also the struggles of going back to work, which made it possible for us to highlight important considerations for care and support of these persons. Capabilities are developed together with others, therefore working towards the same goals while reducing stigma and creating safe and affirmative work environments were identified as crucial components. This study underscores that through an inclusive and collaborative approach involving stakeholders including employers, the social insurance system and the healthcare system, a person with chronic pain can feel supported in developing and cultivating the capabilities necessary to re-engage with work.

## Supporting information

S1 File Interview guide. (PDF)
