# Supplementary material for: Have my back as I get back to work—Experiences of stakeholder support in returning to work after sick leave due to chronic pain: A qualitative interview study
Source: PLoS One. 2024 Oct 23;19(10):e0312478. doi: 10.1371/journal.pone.0312478 (PMC11498695; doi:10.1371/journal.pone.0312478)
Supplement: Attachment — Submitted filename: Response to Reviewers.docx [file pone.0312478.s002.docx]

August 28^th^, 2024

To: Dr Diphale Joyce Mothabeng, Editor

From: Åse Lundin, Corresponding author, [ase.lundin@gu.se](mailto:ase.lundin@gu.se)

**Regarding: Manuscript PONE-D-24-04814**

*Have my back as I get back to work - Experiences of stakeholder support in returning to work after sick leave due to chronic pain: a qualitative interview study*

Revision written by corresponding author Åse Lundin, all authors have reviewed and accepted the revised manuscript.

Dear Dr Diphale Joyce Mothabeng,

Thank you for reviewing our manuscript and giving us the opportunity to revise it, the comments have helped us to improve the manuscript substantially. We have reworked the manuscript according to yours and the reviewers’ comments and suggestions. A point-by-point response to all comments are provided below. All changes can be followed as tracked changes in the manuscript.

We hope that you find our revision of the manuscript and answers to the comments to be satisfactory, otherwise please let us know.

Warm regards,

Corresponding author:

Åse Lundin, MSc, PhD candidate

Sahlgrenska Academy, University of Gothenburg, Sweden

[ase.lundin@gu.se](mailto:ase.lundin@gu.se)

**Journal requirements:**

**1.** Please ensure that your manuscript meets PLOS ONE's style requirements, including those for file naming. The PLOS ONE style templates can be found at:

- <https://journals.plos.org/plosone/s/file?id=wjVg/PLOSOne_formatting_sample_main_body.pdf>
- <https://journals.plos.org/plosone/s/file?id=ba62/PLOSOne_formatting_sample_title_authors_affiliations.pdf>

***Author’s response:*** *Thank you for this observation, we have addressed this issue and revised the manuscript (including the file names) according to the templates provided.*

**2.** We note that you have indicated that there are restrictions to data sharing for this study. PLOS only allows data to be available upon request if there are legal or ethical restrictions on sharing data publicly. For more information on unacceptable data access restrictions, please see <http://journals.plos.org/plosone/s/data-availability#loc-unacceptable-data-access-restrictions>

Before we proceed with your manuscript, please address the following prompts:

**a)** If there are ethical or legal restrictions on sharing a de-identified data set, please explain them in detail (e.g., data contain potentially identifying or sensitive patient information, data are owned by a third-party organization, etc.) and who has imposed them (e.g., a Research Ethics Committee or Institutional Review Board, etc.). Please also provide contact information for a data access committee, ethics committee, or other institutional body to which data requests may be sent.

**b)** If there are no restrictions, please upload the minimal anonymized data set necessary to replicate your study findings to a stable, public repository and provide us with the relevant URLs, DOIs, or accession numbers. For a list of recommended repositories, please see <https://journals.plos.org/plosone/s/recommended-repositories>. You also have the option of uploading the data as Supporting Information files, but we would recommend depositing data directly to a data repository if possible.

We will update your Data Availability statement on your behalf to reflect the information you provide.

***Author’s response:*** *Thank you for this information, we have addressed this issue and revised the Data Availability Statement. In accordance with the decision from the Swedish Ethical Review Authority (Ref. 2020-02491)* *qualitative data from the interviews cannot be shared publicly as it may contain potentially identifying or sensitive participant information, collected from a small group of participants. However, data will be stored for 10 years at the University of Gothenburg to enable review, and data is available upon request from the Institute of Health and Care Sciences at University of Gothenburg. We also added contact information to the institutional body to which data requests may be sent.*

*The data availability statement will be updated in the submission stage and now read as follows:*

In accordance with the decision from the Swedish Ethical Review Authority (Ref. 2020-02491) qualitative data from the interviews cannot be shared publicly as it may contain potentially identifying or sensitive participant information, collected from a small group of participants. All data required to replicate the study’s findings are available in the manuscript through meticulous methodological description, supporting information files and relevant and de-identified quotes and excerpts of the interview transcripts. However, data will be stored for 10 years at the University of Gothenburg to enable review, and data can be made available upon reasonable request from the Institute of Health and Care Sciences at University of Gothenburg, Gothenburg, Sweden, through [vardvetenskap@fhs.gu.se](mailto:vardvetenskap@fhs.gu.se) or by telephone (extension) +46 31-786 10 00.

**Additional Editor Comments:**

Thank you for the manuscript. The reviewers have given a lot of constructive feedback that can be used to improve the document. Kindly address all concerns, especially those of reviewer one with regards to a previous publication.
All the best

***Author’s response:*** *Thank you for considering our manuscript and we agree the reviewers have provided very useful feedback that has greatly improved the manuscript. We have addressed all the concerns and comments and provide a point-by-point response below.*

**Reviewer #1:**

**Comment 1:** Thank you for letting me read your study, which was easy to read. My biggest concern is about research integrity (e.g. honesty and transparency). I see that you have used the same interviews in an earlier study published in BMJ Open. Did you use different parts of the interview guide for different studies? If so, you need to clarify which parts of the interview guide have been analyzed for this particular study. This has consequences for how rich your data is, how well-suited the data is for a phenomenological design, and the limitations you need to discuss. The overall impression I get is that the study results is quite "thin" and superficial, which I did not expect from a phenomenological study. This can be explained by the aforementioned issues. The interviews were quite long but how much of them concerned the results for this particular study...?

***Author’s response:***

*Thank you for your valuable and helpful feedback, which has improved the manuscript significantly. It was part of the study design from the start to have several aims and to design an extensive interview guide that explored various domains of lived experiences of chronic pain, sick leave, disability, activity as well as support while being in the return-to-work process. We believe it might have impaired the interview process if the interviews had been divided into several parts. Therefore, the interview guide (attached to the submission as S1_file) was used in its entirety during the collection of data. In the interviews, the domains were intertwined and thus we used the interviews and guide in their entirety for the studies. The data collected was assessed as both rich and varied, with 14 interviews conducted that ranged in length between 41 and 90 minutes (mean interview length being 70 minutes). This corresponds to about 230 pages when transcribed into text. Hence, we assessed the material as vast, both in quantity but more importantly in quality, which enabled us to continue with the design and aim to conduct two studies based on the same participants and collected data.*

*Regarding the presentation of results, we have addressed this issue and done a revisit to all parts of the structural analysis as well as the comprehensive whole and made subsequent revisions. We have also revised the text in the “Analysis and interpretation of data” section, see page 7, where we have elaborated on the method and how the structural analysis is an early step in the analysis that enables moving on to a more comprehensive understanding. Thus, the structural analysis lies closer to the text and can be viewed as more descriptive in character. A deeper understanding take place first when working with the comprehensive whole, which is the more interpretative part of the results, and in our case leading us to the overarching theme. We have done revisions to try to elaborate on and deepen the result section, see “Results”, page 9-17 including revisions of Figure 1, page 11.*

*We take the matter of research integrity very seriously and please let us know if the above explanation and clarification is not sufficient.*

**Comment 2:** Other, more minor issues are: The rational of the study. You would benefit from describing in the introduction what this type of design can contribute. Currently, you only write that most qualitative studies done are descriptive and that a phenomenological design could increase knowledge. What I am wondering is: in what way and why is it important?

***Author’s response:*** *Thank you for this comment, we agree and have elaborated as well as referenced the rationale of the chosen design and the possible contributions of interpretation in our study, see “introduction” section towards the end of page 4.*

**Comment 3:** I miss the description of the design under the heading "Design." Stating that the study is qualitative is not a sufficient description of the design.

***Author’s response:*** *Thank you for this comment, we agree and have now revised and elaborated the description of the study design, see “design” on page 5.*

**Comment 4:** Your aim is formulated differently in various places; please review this.

***Author’s response:*** *Thank you for your observation, we have now gone through the manuscript and made sure the aim is formulated in the same way in all sections it occurs (page 2 and page 5).*

**Comment 5:** Overall, I think you should consider deepen the results if possible or make it a descriptive study. The study’s focus on support makes it quite unique even if it has a descriptive design. The results are already presented as if it was a descriptive study.

***Author’s response:*** *Thank you for this feedback. Using a phenomenological hermeneutic design we strive to understand the meaning of lived experiences by interpreting the participants narratives. We have addressed the concerns raised and done a revisit to all parts of the data, the structural analysis as well as the comprehensive whole and associated literature, and thereafter made revisions to clarify and elaborate on the interpretative parts of the manuscript. See “Results”, page 9-17 including revisions of Figure 1, page 11.*

*We have also revised the method section “Analysis and interpretation of data” on page 7, where we have elaborated on the methodological aspects regarding the structural analysis being close-to-text and the comprehensive analysis moving away from what the text says and towards interpretation of what living in the world of these participants entails.*

*Again, thank you for all the constructive and valuable feedback and comments, which we believe greatly improved the manuscript.*

**Reviewer #2:**

**Comment 1:** Thank you for the opportunity to review this interesting manuscript. Please see my comments and feedback below, which may help improve the manuscript.

***Author’s response:*** *Thank you for your valuable and helpful feedback, which has improved the manuscript significantly. See our responses to each comment below.*

**Comment 2:** Abstract: Instead of using "purpose and aim," the authors could mention "Background," as they have written it.

***Author’s response:*** *Thank you for your comment, we agree and have revised the title “purpose and aim” to “background” in the abstract section, page 2.*

**Comment 3:** Introduction: It is not clear why this study needs to be conducted; there is a paucity of rationale for the study. In the second paragraph, the authors mentioned vocational rehabilitation. Were the participants in this study undergoing vocational rehabilitation when the data was collected?

***Author’s response:*** *Thank you for your comment. The participants in the study were not currently undergoing vocational rehabilitation, although some of them had in the past. We see that this paragraph in the introduction needed some clarification, it has been revised and we have also elaborated in the text regarding the rationale for the study, see “introduction” on page 3-4.*

**Comment 4:** What aspects or factors are important in the return-to-work process? What has been done and what has not been done in this regard? There have been adequate studies conducted on the return-to-work process that the authors could address.

***Author’s response:*** *Thank you for an important comment, we agree and have revised this section in the introduction and referencing to previous studies, see “introduction” section on page 3 and 4.*

**Comment 5:** Methods - Be consistent with the term "sick leave" (i.e., long-term SL). For example, the authors sometimes mention "prolonged absence" as seen under "Design."

***Author’s response:*** *Thank you for this observation, we certainly agree consistency is key and we have now revised the manuscript to make sure it is the same terminology and using “sick leave” throughout.*

**Comment 6:** Under "Participants and Setting," mention the duration of SL. The information in this section is sparse, so I recommend adding more details about the sampling procedure and how participants were chosen for the study. Include the exclusion criteria.

***Author’s response:*** *Thank you for your comment, we agree the information in this section was sparse and we have added more information about the sampling procedure. We opted on having only inclusion criteria for this study, the ones fulfilling the criteria were eligible to participate, thus we did not have exclusion criteria. See the revised section "Participants and Setting" page 5 and 6.*

*Unfortunately we do not have data on the duration of the participants’ sick leave. At the time of data collection, we assessed that the collection of data on age, gender, education, pain condition, duration of pain and work status was sufficient. We have elaborated on this matter as a limitation in the “Methodological considerations and limitations” section, page 23. We do acknowledge that data on the duration of sick leave as part of participant characteristics would add an important piece of information, and we take this knowledge with us into further studies and publications.*

**Comment 7:** One concern for this study is the age range. If someone is 80 years old and retired, how did the authors obtain experiences on return-to-work from that person? This is a significant limitation of the study. It is unclear what steps were taken to avoid this kind of bias/error.

***Author’s response:*** *Thank you for your comment. We agree it is a limitation, as the participant had retired and was currently not in the process of going back to work. However the participant had long time past experience of sick leave and the return to work-process throughout her working life. Using a phenomenological hermeneutic method we want to gain as many perspectives and experiences as possible to achieve narratives regarding the phenomenon that is both rich and varied. We did not want to* *exclude this participant as we found she had very important reflections looking back at her return-to-work process that gave insights we would otherwise have missed out upon.*

*The participant met our inclusion criteria, as we did not exclude by age and having both current and previous experiences of sick leave was deemed eligible. We have clarified the sampling/inclusion process and criteria in “participants and setting” section page 5 and 6. We have also added reflections on this significant limitation in the discussion section, page 23. Thank you for making us aware of this issue.*

**Comment 8:** Using audio interviews for a qualitative study is somewhat unconventional for dealing with the data. Since the authors have already done so, they should include a reference to support this approach.
***Author’s response:*** *Thank you for your comment. We want to clarify how we worked with the collection and analysis of data. The interviews were recorded on audio using a dictaphone, this was done in order to be able to transcribe them verbatim into text afterwards, which is a commonly used procedure in qualitative research. Thus the transcribed text is considered the actual data material collected and was used in the analysis. We have clarified this process in the manuscript, see section “data collection”, page 6.*

**Comment 9:** Results: The only question I have is how the authors came up with the theme "Have my back as I get back to work." This phrase is in the title, so how could it also be a theme? So far, I noted that the authors formulated six themes by condensing meaning units.

***Author’s response:*** *Thank you for your question. The theme "Have my back as I get back to work" was identified during the process of comprehensive understanding of the data collected, a result reached after considering the naïve reading, structural analysis and our pre-understanding. Additionally, we consulted relevant literature to enhance our interpretation and achieve an in-depth understanding of what it is like living in the world that the text opens up. We have clarified this process in section “analysis and interpretation of data”, page 7 and under “Comprehensive understanding”, page 16.*

*Using phenomenological hermeneutics is a constant back-and-forth movement between the parts and the whole i.e. the structural, the naïve and the comprehensive understanding of the text. Thus, as we saw this interpretation emerge and that it was running as a continuing element through all parts of the results we decided it could be regarded as a theme. As the overarching theme, to highlight this and clarify its role, we also revised the figure, see revised figure 1, page 11. Lastly, to make the study and its title eye-catching we decided to put the theme into the title of the manuscript. It is not uncommon to have theme names or interview quotes or excerpts in the title of qualitative studies. However, if this explanation is not sufficient, or you strongly advise us to revise the title, please let us know.*

*Again, thank you for all the constructive and valuable feedback and comments, which we believe have significantly improved the manuscript.*
