# Supplementary material for: Have my back as I get back to work—Experiences of stakeholder support in returning to work after sick leave due to chronic pain: A qualitative interview study
Source: PLoS One. 2024 Oct 23;19(10):e0312478. doi: 10.1371/journal.pone.0312478 (PMC11498695; doi:10.1371/journal.pone.0312478)
Supplement: Peer review history

# Peer review: Decision Letter 0

**DOI:** 10.1371/journal.pone.0312478.r001

**Authors:** Diphale Joyce Mothabeng

6 Aug 2024

PONE-D-24-04814Have my back as I get back to work - Experiences of stakeholder support in returning to work after sick leave due to chronic pain: A qualitative studyPLOS ONE

Dear Dr. Lundin,

Thank you for submitting your manuscript to PLOS ONE. After careful consideration, we feel that it has merit but does not fully meet PLOS ONE’s publication criteria as it currently stands. Therefore, we invite you to submit a revised version of the manuscript that addresses the points raised during the review process.

Please submit your revised manuscript by Sep 20 2024 11:59PM. If you will need more time than this to complete your revisions, please reply to this message or contact the journal office at plosone@plos.org. When you're ready to submit your revision, log on to https://www.editorialmanager.com/pone/ and select the 'Submissions Needing Revision' folder to locate your manuscript file.

Please include the following items when submitting your revised manuscript:

- A rebuttal letter that responds to each point raised by the academic editor and reviewer(s). You should upload this letter as a separate file labeled 'Response to Reviewers'.
- A marked-up copy of your manuscript that highlights changes made to the original version. You should upload this as a separate file labeled 'Revised Manuscript with Track Changes'.
- An unmarked version of your revised paper without tracked changes. You should upload this as a separate file labeled 'Manuscript'.

If you would like to make changes to your financial disclosure, please include your updated statement in your cover letter. Guidelines for resubmitting your figure files are available below the reviewer comments at the end of this letter.

If applicable, we recommend that you deposit your laboratory protocols in protocols.io to enhance the reproducibility of your results. Protocols.io assigns your protocol its own identifier (DOI) so that it can be cited independently in the future. For instructions see: https://journals.plos.org/plosone/s/submission-guidelines#loc-laboratory-protocols. Additionally, PLOS ONE offers an option for publishing peer-reviewed Lab Protocol articles, which describe protocols hosted on protocols.io. Read more information on sharing protocols at https://plos.org/protocols?utm_medium=editorial-email&utm_source=authorletters&utm_campaign=protocols.

We look forward to receiving your revised manuscript.

Kind regards,

Diphale Joyce Mothabeng, PhD

Academic Editor

PLOS ONE

Journal requirements:

When submitting your revision, we need you to address these additional requirements.

1. Please ensure that your manuscript meets PLOS ONE's style requirements, including those for file naming. The PLOS ONE style templates can be found at

https://journals.plos.org/plosone/s/file?id=wjVg/PLOSOne_formatting_sample_main_body.pdf and

https://journals.plos.org/plosone/s/file?id=ba62/PLOSOne_formatting_sample_title_authors_affiliations.pdf

2. We note that you have indicated that there are restrictions to data sharing for this study. PLOS only allows data to be available upon request if there are legal or ethical restrictions on sharing data publicly. For more information on unacceptable data access restrictions, please see http://journals.plos.org/plosone/s/data-availability#loc-unacceptable-data-access-restrictions.

Before we proceed with your manuscript, please address the following prompts:

a) If there are ethical or legal restrictions on sharing a de-identified data set, please explain them in detail (e.g., data contain potentially identifying or sensitive patient information, data are owned by a third-party organization, etc.) and who has imposed them (e.g., a Research Ethics Committee or Institutional Review Board, etc.). Please also provide contact information for a data access committee, ethics committee, or other institutional body to which data requests may be sent.

b) If there are no restrictions, please upload the minimal anonymized data set necessary to replicate your study findings to a stable, public repository and provide us with the relevant URLs, DOIs, or accession numbers. For a list of recommended repositories, please see

https://journals.plos.org/plosone/s/recommended-repositories. You also have the option of uploading the data as Supporting Information files, but we would recommend depositing data directly to a data repository if possible.

We will update your Data Availability statement on your behalf to reflect the information you provide.

Additional Editor Comments:

Thank you for the manuscript. The reviewers have given a lot of constructive feedback that can be used to improve the document. Kindly address all concerns, especially those of reviewer one with regards to a previous publication.

All the best

[Note: HTML markup is below. Please do not edit.]

Reviewers' comments:

Reviewer's Responses to Questions

**Comments to the Author**

1. Is the manuscript technically sound, and do the data support the conclusions?

The manuscript must describe a technically sound piece of scientific research with data that supports the conclusions. Experiments must have been conducted rigorously, with appropriate controls, replication, and sample sizes. The conclusions must be drawn appropriately based on the data presented. 

Reviewer #1: No

Reviewer #2: Yes

**********

2. Has the statistical analysis been performed appropriately and rigorously? 

Reviewer #1: N/A

Reviewer #2: N/A

**********

3. Have the authors made all data underlying the findings in their manuscript fully available?

The PLOS Data policy requires authors to make all data underlying the findings described in their manuscript fully available without restriction, with rare exception (please refer to the Data Availability Statement in the manuscript PDF file). The data should be provided as part of the manuscript or its supporting information, or deposited to a public repository. For example, in addition to summary statistics, the data points behind means, medians and variance measures should be available. If there are restrictions on publicly sharing data—e.g. participant privacy or use of data from a third party—those must be specified.

Reviewer #1: No

Reviewer #2: Yes

**********

4. Is the manuscript presented in an intelligible fashion and written in standard English?

PLOS ONE does not copyedit accepted manuscripts, so the language in submitted articles must be clear, correct, and unambiguous. Any typographical or grammatical errors should be corrected at revision, so please note any specific errors here.

Reviewer #1: Yes

Reviewer #2: Yes

**********

5. Review Comments to the Author

Please use the space provided to explain your answers to the questions above. You may also include additional comments for the author, including concerns about dual publication, research ethics, or publication ethics. (Please upload your review as an attachment if it exceeds 20,000 characters)

Reviewer #1: Thank you for letting me read your study, which was easy to read.

My biggest concern is about research integrity (e.g. honesty and transparency). I see that you have used the same interviews in an earlier study published in BMJ Open. Did you use different parts of the interview guide for different studies? If so, you need to be clarify which parts of the interview guide have been analyzed for this particular study. This has consequences for how rich your data is, how well-suited the data is for a phenomenological design, and the limitations you need to discuss. The overall impression I get is that the study results is quite "thin" and superficial, which I did not expect from a phenomenological study. This can be explained by the aforementioned issues. The interviews were quite long but how much of them concerned the results for this particular study...?

Other, more minor issues are:

The rational of the study. You would benefit from describing in the introduction what this type of design can contribute. Currently, you only write that most qualitative studies done are descriptive and that a phenomenological design could increase knowledge. What I am wondering is: in what way and why is it important?

I miss the description of the design under the heading "Design." Stating that the study is qualitative is not a sufficient description of the design.

Your aim is formulated differently in various places; please review this.

Overall, I think you should consider deepen the results if possible or make it a descriptive study. The studys focus on support makes it quite unique even if it has a descriptive design. The results are already presented as if it was a descriptive study.

Reviewer #2: Thank you for the opportunity to review this interesting manuscript. Please see my comments and feedback below, which may help improve the manuscript.

Abstract:

Instead of using "purpose and aim," the authors could mention "Background," as they have written it.

Introduction:

-It is not clear why this study needs to be conducted; there is a paucity of rationale for the study. In the second paragraph, the authors mentioned vocational rehabilitation. Were the participants in this study undergoing vocational rehabilitation when the data was collected?

-What aspects or factors are important in the return-to-work process? What has been done and what has not been done in this regard? There have been adequate studies conducted on the return-to-work process that the authors could address.

Methods:

- Be consistent with the term "sick leave" (i.e., long-term SL). For example, the authors sometimes mention "prolonged absence" as seen under "Design."

- Under "Participants and Setting," mention the duration of SL. The information in this section is sparse, so I recommend adding more details about the sampling procedure and how participants were chosen for the study. Include the exclusion criteria.

- One concern for this study is the age range. If someone is 80 years old and retired, how did the authors obtain experiences on return-to-work from that person? This is a significant limitation of the study. It is unclear what steps were taken to avoid this kind of bias/error.

- Using audio interviews for a qualitative study is somewhat unconventional for dealing with the data. Since the authors have already done so, they should include a reference to support this approach.

Results:

The only question I have is how the authors came up with the theme "Have my back as I get back to work." This phrase is in the title, so how could it also be a theme? So far, I noted that the authors formulated six themes by condensing meaning units.

**********

6. PLOS authors have the option to publish the peer review history of their article (what does this mean?). If published, this will include your full peer review and any attached files.

If you choose “no”, your identity will remain anonymous but your review may still be made public.

**Do you want your identity to be public for this peer review?** For information about this choice, including consent withdrawal, please see our Privacy Policy.

Reviewer #1: No

Reviewer #2: **Yes: **Dr. Mamunur Rashid

Assistant Professor

**********

[NOTE: If reviewer comments were submitted as an attachment file, they will be attached to this email and accessible via the submission site. Please log into your account, locate the manuscript record, and check for the action link "View Attachments". If this link does not appear, there are no attachment files.]

While revising your submission, please upload your figure files to the Preflight Analysis and Conversion Engine (PACE) digital diagnostic tool, https://pacev2.apexcovantage.com/. PACE helps ensure that figures meet PLOS requirements. To use PACE, you must first register as a user. Registration is free. Then, login and navigate to the UPLOAD tab, where you will find detailed instructions on how to use the tool. If you encounter any issues or have any questions when using PACE, please email PLOS at figures@plos.org. Please note that Supporting Information files do not need this step.

# Author comment: Author response to Decision Letter 0

**DOI:** 10.1371/journal.pone.0312478.r002

13 Sep 2024

To: Dr Diphale Joyce Mothabeng, Editor 

From: Åse Lundin, Corresponding author, ase.lundin@gu.se

Regarding: Manuscript PONE-D-24-04814

Have my back as I get back to work - Experiences of stakeholder support in returning to work after sick leave due to chronic pain: a qualitative interview study

Revision written by corresponding author Åse Lundin, all authors have reviewed and accepted the revised manuscript.

Thank you for reviewing our manuscript and giving us the opportunity to revise it, the comments have helped us to improve the manuscript substantially. We have reworked the manuscript according to yours and the reviewers’ comments and suggestions. A point-by-point response to all comments are provided below. All changes can be followed as tracked changes in the manuscript. 

We hope that you find our revision of the manuscript and answers to the comments to be satisfactory, otherwise please let us know. 

Warm regards, 

Corresponding author:

Åse Lundin, MSc, PhD candidate 

Sahlgrenska Academy, University of Gothenburg, Sweden

ase.lundin@gu.se

Journal requirements:

1. Please ensure that your manuscript meets PLOS ONE's style requirements, including those for file naming. The PLOS ONE style templates can be found at:

• https://journals.plos.org/plosone/s/file?id=wjVg/PLOSOne_formatting_sample_main_body.pdf

• https://journals.plos.org/plosone/s/file?id=ba62/PLOSOne_formatting_sample_title_authors_affiliations.pdf

Author’s response: Thank you for this observation, we have addressed this issue and revised the manuscript (including the file names) according to the templates provided. 

2. We note that you have indicated that there are restrictions to data sharing for this study. PLOS only allows data to be available upon request if there are legal or ethical restrictions on sharing data publicly. For more information on unacceptable data access restrictions, please see http://journals.plos.org/plosone/s/data-availability#loc-unacceptable-data-access-restrictions

Before we proceed with your manuscript, please address the following prompts:

a) If there are ethical or legal restrictions on sharing a de-identified data set, please explain them in detail (e.g., data contain potentially identifying or sensitive patient information, data are owned by a third-party organization, etc.) and who has imposed them (e.g., a Research Ethics Committee or Institutional Review Board, etc.). Please also provide contact information for a data access committee, ethics committee, or other institutional body to which data requests may be sent.

b) If there are no restrictions, please upload the minimal anonymized data set necessary to replicate your study findings to a stable, public repository and provide us with the relevant URLs, DOIs, or accession numbers. For a list of recommended repositories, please see https://journals.plos.org/plosone/s/recommended-repositories. You also have the option of uploading the data as Supporting Information files, but we would recommend depositing data directly to a data repository if possible.

We will update your Data Availability statement on your behalf to reflect the information you provide.

Author’s response: Thank you for this information, we have addressed this issue and revised the Data Availability Statement. In accordance with the decision from the Swedish Ethical Review Authority (Ref. 2020-02491) qualitative data from the interviews cannot be shared publicly as it may contain potentially identifying or sensitive participant information, collected from a small group of participants. However, data will be stored for 10 years at the University of Gothenburg to enable review, and data is available upon request from the Institute of Health and Care Sciences at University of Gothenburg. We also added contact information to the institutional body to which data requests may be sent.

The data availability statement will be updated in the submission stage and now read as follows:

In accordance with the decision from the Swedish Ethical Review Authority (Ref. 2020-02491) qualitative data from the interviews cannot be shared publicly as it may contain potentially identifying or sensitive participant information, collected from a small group of participants. All data required to replicate the study’s findings are available in the manuscript through meticulous methodological description, supporting information files and relevant and de-identified quotes and excerpts of the interview transcripts. However, data will be stored for 10 years at the University of Gothenburg to enable review, and data can be made available upon reasonable request from the Institute of Health and Care Sciences at University of Gothenburg, Gothenburg, Sweden, through vardvetenskap@fhs.gu.se or by telephone (extension) +46 31-786 10 00.

Additional Editor Comments:

Thank you for the manuscript. The reviewers have given a lot of constructive feedback that can be used to improve the document. Kindly address all concerns, especially those of reviewer one with regards to a previous publication.

All the best

Author’s response: Thank you for considering our manuscript and we agree the reviewers have provided very useful feedback that has greatly improved the manuscript. We have addressed all the concerns and comments and provide a point-by-point response below. 

Reviewer #1: 

Comment 1: Thank you for letting me read your study, which was easy to read. My biggest concern is about research integrity (e.g. honesty and transparency). I see that you have used the same interviews in an earlier study published in BMJ Open. Did you use different parts of the interview guide for different studies? If so, you need to clarify which parts of the interview guide have been analyzed for this particular study. This has consequences for how rich your data is, how well-suited the data is for a phenomenological design, and the limitations you need to discuss. The overall impression I get is that the study results is quite "thin" and superficial, which I did not expect from a phenomenological study. This can be explained by the aforementioned issues. The interviews were quite long but how much of them concerned the results for this particular study...?

Author’s response: 

Thank you for your valuable and helpful feedback, which has improved the manuscript significantly. It was part of the study design from the start to have several aims and to design an extensive interview guide that explored various domains of lived experiences of chronic pain, sick leave, disability, activity as well as support while being in the return-to-work process. We believe it might have impaired the interview process if the interviews had been divided into several parts. Therefore, the interview guide (attached to the submission as S1_file) was used in its entirety during the collection of data. In the interviews, the domains were intertwined and thus we used the interviews and guide in their entirety for the studies. The data collected was assessed as both rich and varied, with 14 interviews conducted that ranged in length between 41 and 90 minutes (mean interview length being 70 minutes). This corresponds to about 230 pages when transcribed into text. Hence, we assessed the material as vast, both in quantity but more importantly in quality, which enabled us to continue with the design and aim to conduct two studies based on the same participants and collected data. 

Regarding the presentation of results, we have addressed this issue and done a revisit to all parts of the structural analysis as well as the comprehensive whole and made subsequent revisions. We have also revised the text in the “Analysis and interpretation of data” section, see page 7, where we have elaborated on the method and how the structural analysis is an early step in the analysis that enables moving on to a more comprehensive understanding. Thus, the structural analysis lies closer to the text and can be viewed as more descriptive in character. A deeper understanding take place first when working with the comprehensive whole, which is the more interpretative part of the results, and in our case leading us to the overarching theme. We have done revisions to try to elaborate on and deepen the result section, see “Results”, page 9-17 including revisions of Figure 1, page 11.

We take the matter of research integrity very seriously and please let us know if the above explanation and clarification is not sufficient. 

Comment 2: Other, more minor issues are: The rational of the study. You would benefit from describing in the introduction what this type of design can contribute. Currently, you only write that most qualitative studies done are descriptive and that a phenomenological design could increase knowledge. What I am wondering is: in what way and why is it important?

Author’s response: Thank you for this comment, we agree and have elaborated as well as referenced the rationale of the chosen design and the possible contributions of interpretation in our study, see “introduction” section towards the end of page 4. 

Comment 3: I miss the description of the design under the heading "Design." Stating that the study is qualitative is not a sufficient description of the design.

Author’s response: Thank you for this comment, we agree and have now revised and elaborated the description of the study design, see “design” on page 5. 

Comment 4: Your aim is formulated differently in various places; please review this.

Author’s response: Thank you for your observation, we have now gone through the manuscript and made sure the aim is formulated in the same way in all sections it occurs (page 2 and page 5). 

Comment 5: Overall, I think you should consider deepen the results if possible or make it a descriptive study. The study’s focus on support makes it quite unique even if it has a descriptive design. The results are already presented as if it was a descriptive study.

Author’s response: Thank you for this feedback. Using a phenomenological hermeneutic design we strive to understand the meaning of lived experiences by interpreting the participants narratives. We have addressed the concerns raised and done a revisit to all parts of the data, the structural analysis as well as the comprehensive whole and associated literature, and thereafter made revisions to clarify and elaborate on the interpretative parts of the manuscript. See “Results”, page 9-17 including revisions of Figure 1, page 11.

We have also revised the method section “Analysis and interpretation of data” on page 7, where we have elaborated on the methodological aspects regarding the structural analysis being close-to-text and the comprehensive analysis moving away from what the text says and towards interpretation of what living in the world of these participants entails.

Again, thank you for all the constructive and valuable feedback and comments, which we believe greatly improved the manuscript. 

Reviewer #2: 

Comment 1: Thank you for the opportunity to review this interesting manuscript. Please see my comments and feedback below, which may help improve the manuscript.

Author’s response: Thank you for your valuable and helpful feedback, which has improved the manuscript significantly. See our responses to each comment below. 

Comment 2: Abstract: Instead of using "purpose and aim," the authors could mention "Background," as they have written it.

Author’s response: Thank you for your comment, we agree and have revised the title “purpose and aim” to “background” in the abstract section, page 2. 

Comment 3: Introduction: It is not clear why this study needs to be conducted; there is a paucity of rationale for the study. In the second paragraph, the authors mentioned vocational rehabilitation. Were the participants in this study undergoing vocational rehabilitation when the data was collected?

Author’s response: Thank you for your comment. The participants in the study were not currently undergoing vocational rehabilitation, although some of them had in the past. We see that this paragraph in the introduction needed some clarification, it has been revised and we have also elaborated in the text regarding the rationale for the study, see “introduction” on page 3-4.

Comment 4: What aspects or factors are important in the return-to-work process? What has been done and what has not been done in this regard? There have been adequate studies conducted on the return-to-work process that the authors could address.

Author’s response: Thank you for an important comment, we agree and have revised this section in the introduction and referencing to previous studies, see “introduction” section on page 3 and 4.

Comment 5: Methods - Be consistent with the term "sick leave" (i.e., long-term SL). For example, the authors sometimes mention "prolonged absence" as seen under "Design."

Author’s response: Thank you for this observation, we certainly agree consistency is key and we have now revised the manuscript to make sure it is the same terminology and using “sick leave” throughout. 

Comment 6: Under "Participants and Setting," mention the duration of SL. The information in this section is sparse, so I recommend adding more details about the sampling procedure and how participants were chosen for the study. Include the exclusion criteria.

Author’s response: Thank you for your comment, we agree the information in this section was sparse and we have added more information about the sampling procedure. We opted on having only inclusion criteria for this study, the ones fulfilling the criteria were eligible to participate, thus we did not have exclusion criteria. See the revised section "Participants and Setting" page 5 and 6. 

Unfortunately we do not have data on the duration of the participants’ sick leave. At the time of data collection, we assessed that the collection of data on age, gender, education, pain condition, duration of pain and work status was sufficient. We have elaborated on this matter as a limitation in the “Methodological considerations and limitations” section, page 23. We do acknowledge that data on the duration of sick leave as part of participant characteristics would add an important piece of information, and we take this knowledge with us into further studies and publications. 

Comment 7: One concern for this study is the age range. If someone is 80 years old and retired, how did the authors obtain experiences on return-to-work from that person? This is a significant limitation of the study. It is unclear what steps were taken to avoid this kind of bias/error.

Author’s response: Thank you for your comment. We agree it is a limitation, as the participant had retired and was currently not in the process of going back to work. However the participant had long time past experience of sick leave and the return to work-process throughout her working life. Using a phenomenological hermeneutic method we want to gain as many perspectives and experiences as possible to achieve narratives regarding the phenomenon that is both rich and varied. We did not want to exclude this participant as we found she had very important reflections looking back at her return-to-work process that gave insights we would otherwise have missed out upon. 

The participant met our inclusion criteria, as we did not exclude by age and having both current and previous experiences of sick leave was deemed eligible. We have clarified the sampling/inclusion process and criteria in “participants and setting” section page 5 and 6. We have also added reflections on this significant limitation in the discussion section, page 23. Thank you for making us aware of this issue. 

Comment 8: Using audio interviews for a qualitative study is somewhat unconventional for dealing with the data. Since the authors have already done so, they should include a reference to support this approach.

Author’s response: Thank you for your comment. We want to clarify how we worked with the collection and analysis of data. The interviews were recorded on audio using a dictaphone, this was done in order to be able to transcribe them verbatim into text afterwards, which is a commonly used procedur

Attachment Submitted filename: Response to Reviewers.docx

# Editor report: Decision Letter 1

**DOI:** 10.1371/journal.pone.0312478.r003

**Authors:** Diphale Joyce Mothabeng

8 Oct 2024

Have my back as I get back to work - Experiences of stakeholder support in returning to work after sick leave due to chronic pain: a qualitative interview study

PONE-D-24-04814R1

Dear Dr. Lundin,

We’re pleased to inform you that your manuscript has been judged scientifically suitable for publication and will be formally accepted for publication once it meets all outstanding technical requirements.

Within one week, you’ll receive an e-mail detailing the required amendments. When these have been addressed, you’ll receive a formal acceptance letter and your manuscript will be scheduled for publication.

An invoice will be generated when your article is formally accepted. Please note, if your institution has a publishing partnership with PLOS and your article meets the relevant criteria, all or part of your publication costs will be covered. Please make sure your user information is up-to-date by logging into Editorial Manager at Editorial Manager® and clicking the ‘Update My Information' link at the top of the page. If you have any questions relating to publication charges, please contact our Author Billing department directly at authorbilling@plos.org.

If your institution or institutions have a press office, please notify them about your upcoming paper to help maximize its impact. If they’ll be preparing press materials, please inform our press team as soon as possible -- no later than 48 hours after receiving the formal acceptance. Your manuscript will remain under strict press embargo until 2 pm Eastern Time on the date of publication. For more information, please contact onepress@plos.org.

Kind regards,

Diphale Joyce Mothabeng, PhD

Academic Editor

PLOS ONE

Additional Editor Comments (optional):

Thank you for addressing the reviewer comments.

The journal will revert back to you on the way forward.

Reviewers' comments:

# Editor report: Acceptance letter

**DOI:** 10.1371/journal.pone.0312478.r004

**Authors:** Diphale Joyce Mothabeng

11 Oct 2024

PONE-D-24-04814R1 

PLOS ONE

Dear Dr. Lundin, 

I'm pleased to inform you that your manuscript has been deemed suitable for publication in PLOS ONE. Congratulations! Your manuscript is now being handed over to our production team.

At this stage, our production department will prepare your paper for publication. This includes ensuring the following:

* All references, tables, and figures are properly cited

* All relevant supporting information is included in the manuscript submission,

* There are no issues that prevent the paper from being properly typeset

If revisions are needed, the production department will contact you directly to resolve them. If no revisions are needed, you will receive an email when the publication date has been set. At this time, we do not offer pre-publication proofs to authors during production of the accepted work. Please keep in mind that we are working through a large volume of accepted articles, so please give us a few weeks to review your paper and let you know the next and final steps. 

Lastly, if your institution or institutions have a press office, please let them know about your upcoming paper now to help maximize its impact. If they'll be preparing press materials, please inform our press team within the next 48 hours. Your manuscript will remain under strict press embargo until 2 pm Eastern Time on the date of publication. For more information, please contact onepress@plos.org.

If we can help with anything else, please email us at customercare@plos.org.

Thank you for submitting your work to PLOS ONE and supporting open access. 

Kind regards, 

PLOS ONE Editorial Office Staff

on behalf of

Dr. Diphale Joyce Mothabeng 

Academic Editor

PLOS ONE
